# A Novel Inhibitor against the Bromodomain Protein 1 of the Malaria Pathogen *Plasmodium Falciparum*

**DOI:** 10.1002/cmdc.202500024

**Published:** 2025-04-07

**Authors:** Marius Amann, Robin Warstat, Kay Kristin Rechten, Philip Theuer, Magdalena Schustereder, Sophie Clavey, Bernhard Breit, Oliver Einsle, Martin Hügle, Michaela Petter, Stefan Günther

**Affiliations:** ^1^ Institut für Pharmazeutische Wissenschaften Albert‐Ludwigs‐Universität Freiburg Hermann‐Herder‐Str. 9 79104 Freiburg im Breisgau Germany; ^2^ Institut für Biochemie Albert‐Ludwigs‐Universität Freiburg Alberstrasse 21 79104 Freiburg im Breisgau Germany; ^3^ Institut für Organische Chemie und Biochemie Albert‐Ludwigs‐Universität Freiburg Albertstrasse 21 79104 Freiburg im Breisgau Germany; ^4^ Mikrobiologisches Institut ‐ Klinische Mikrobiologie, Immunologie und Hygiene Universitätsklinikum Erlangen and Friedrich‐Alexander‐Universität (FAU) Erlangen‐Nürnberg Wasserturmstr. 3‐5 91054 Erlangen Germany

**Keywords:** Antiprotozoal agents, bromodomain, drug discovery, inhibitors, Plasmodium

## Abstract

The rise of drug resistances in malaria necessitates the exploration of novel therapeutic strategies. Targeting epigenetic pathways could open new, promising treatment avenues. In this study, the focus is on the essential Bromodomain protein 1 (*Pf*BDP1) of the malaria pathogen *Plasmodium falciparum.* Utilizing the pan‐selective bromodomain inhibitor MPM6, a potent initial hit is identified and it is subsequently developed into a nanomolar binder. Through a combination of virtual docking, isothermal titration calorimetry, and X‐ray crystallography, the molecular interactions of the new inhibitors with the bromodomain (BRD) of the protein (*Pf*BDP1‐BRD) are elucidated. The findings include the first co‐crystallized inhibitors with the structures of *Pf*BRD1‐BRD as well as the bromodomain of the close homologous protein of *Plasmodium vivax* (*Pv*BDP1‐BRD). The structures provide new insights into their binding mechanisms. Further validation using conditional knockdown of *Pf*BDP1 in *P. falciparum* demonstrates parasite sensitivity to the inhibitor, underscoring its potential in a targeted therapeutic approach against malaria.

## Graphical abstract

This article presents the development of a first‐in‐class inhibitor targeting the bromodomain protein *Pf*BDP1 in *Plasmodium falciparum*, the deadliest variant of the malaria‐causing *Plasmodium* parasites. Through structure‐based drug design, the inhibitor interactions were characterized via X‐ray crystallography and isothermal titration calorimetry. Conditional knockdown and ChIP assays validate *Pf*BDP1 inhibition, highlighting RMM23 as a promising starting point in malaria drug discovery

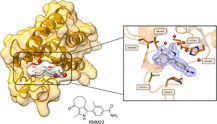

## 1 Introduction

Malaria, caused by the eukaryotic parasite *Plasmodium spec.*, is a major public health issue, with ≈249 million infected and 608,000 deaths worldwide reported in 2022.^[^ ^1^ ^]^ The majority of casualties are attributed to *Plasmodium falciparum* (*P. falciparum*), the most lethal variant of the *Plasmodium* parasites. Despite intensive efforts to control the disease, little progress has been made in recent years toward reducing malaria burden and death; indeed, drug resistances have developed against the two most common drugs (artemisinin‐based combination therapies and atovaquone–proguanil), highlighting the need for new therapeutic approaches.^[^ ^2^, ^3^, ^4^ ^]^ Recent studies see potential in the disruption of epigenetic pathways.^[^ ^5^, ^6^, ^7^, ^8^ ^]^ Possible targets are the nine predicted bromodomain (BRD) proteins, among which *Pf*BDP1 has been identified as essential in the blood stage of *P. falciparum*.^[^ ^9^, ^10^, ^11^ ^]^ *Pf*BDP1 is involved in regulating erythrocyte invasion genes and conditional knockdown (KD) of *Pf*BDP1 lead to a significantly reduced multiplication rate during the intraerythrocytic development cycle (IDC).^[^ ^12^ ^]^ It remains elusive whether this effect could also be induced by directly targeting the BRD through small molecule inhibition. A recent structural and binding analysis of *Pf*BDP1‐BRD revealed that a tetra‐acetylated histone H4 peptide showed highest binding affinity; however, there is no co‐crystallized structure with a natural ligand or inhibitor.^[^ ^13^ ^]^

Within the present study, we address the effect of inhibiting the *Pf*BDP1‐BRD using small molecule inhibitors based on the recently published human pan‐selective BRD inhibitor (BRDi) MPM6.^[^ ^14^ ^]^ We first simplified the inhibitor to identify relevant parts for target affinity. We then introduced new modifications to increase affinity, which was achieved by rational molecule design in combination with molecular modeling, chemical synthesis, isothermal titration calorimetry (ITC), and X‐ray crystallography (XRD). With the newly developed inhibitors, we succeeded in generating co‐crystal structures of *Pf*BDP1‐BRD as well as *Pv*BDP1‐BRD, the BRD of *Plasmodium vivax*, showing detailed insights into the molecular interactions. Subsequently, the best inhibitors were tested for activity against various *P*. *falciparum* strains during their IDC. Conditional KD of *Pf*BDP1 was used for target validation, demonstrating differential sensitivity to one of the inhibitors when limiting *Pf*BDP1 expression. Additionally, in the presence of this inhibitor, *Pf*BDP1 was displaced from its target genes, as shown by chromatin immunoprecipitation (ChIP).

## 2 Results and Discussion

**Figure 1:**
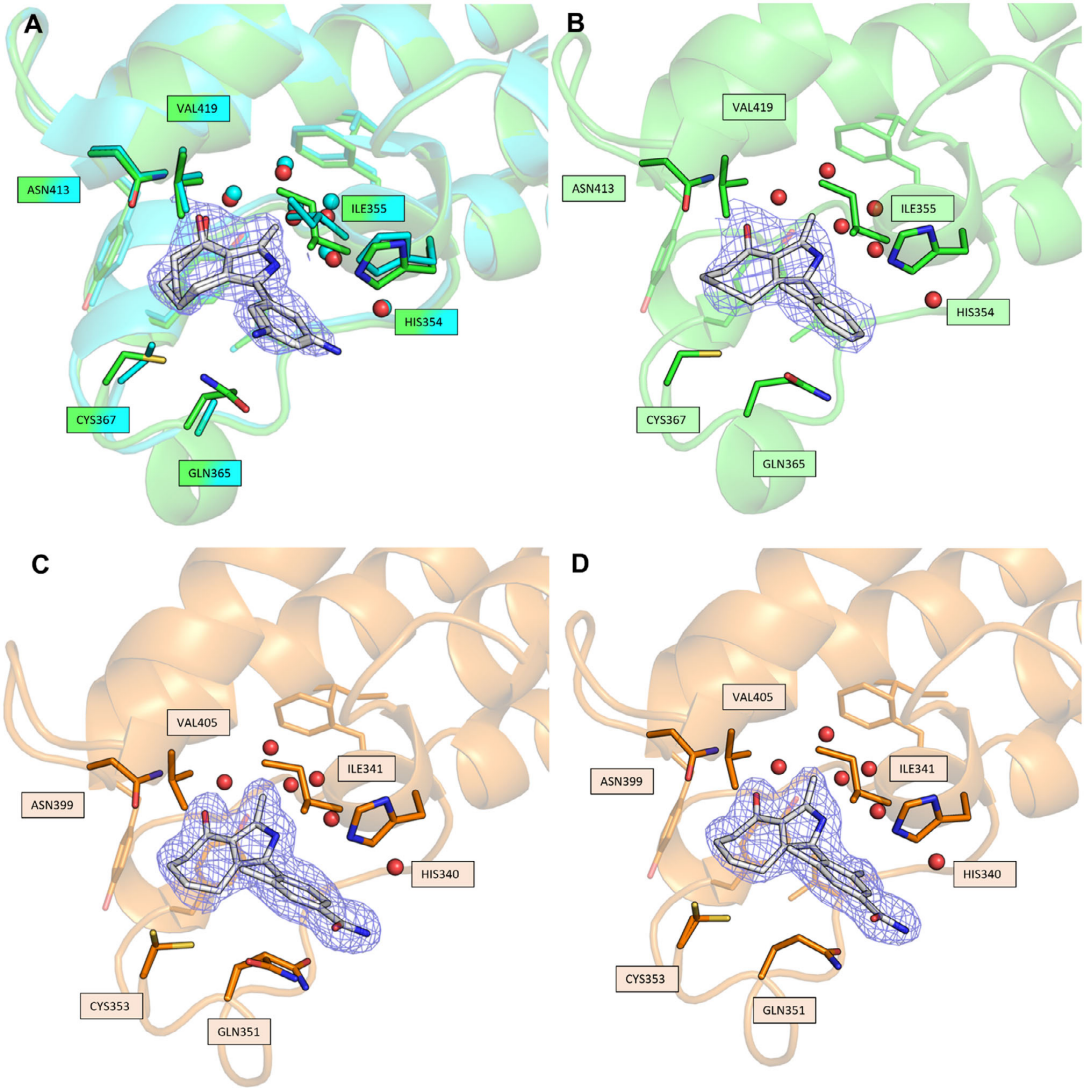
X‐ray structures of *Pf*BDP1‐BRD and *Pv*BDP1‐BRD in complex with our designed ligands alongside their 2mFo‐DFc composite omit maps contoured at 1.0 σ. A) *Pf*BDP1‐BRD (green) complexed with MPM2 (white) overlayed with apo‐*Pf*BDP1‐BRD (teal; PDB ID:7M97). The conformation of the residues inside the BPs are almost identical except for showing an additional conformation for CYS367 and a side chain flip for ILE355. B) *Pf*BDP1‐BRD (green) complexed with RMM2 (white). The *ortho*‐methyl group forms hydrophobic interactions with the side chains of ILE355, the gatekeeper Val419, and the seven‐membered ring. C) *Pv*BDP1‐BRD (orange) complexed with RMM23 (white). Two conformations of GLN351 are visible. D) *Pv*BDP1‐BRD (orange) complexed with RMM25 (white). The additional *ortho*‐methyl group points toward the inner pocket.

First, we determined the affinity using ITC of the pan‐selective BRDi MPM6 for *Pf*BDP1‐BRD (4.53 μM, **Table** **1**), which thus represented our first hit. Subsequently, we generated simplified variants of MPM6 to verify the contribution of the chloride and amino groups. Both resulting ligands showed slightly decreased affinity, with the contribution of the chloride (RMM1, 5.8 μM, Table 1) found to be more important than that of the amino group (MPM2, 6.7 μM). Removal of both groups led to a more pronounced reduction in affinity (MPM3, 10 μM, Table 1). The co‐crystal structure of MPM2 with *Pf*BDP1‐BRD (Protein Data Bank Identifier, PDB ID: 9HHC) is consistent with the apo structure (PDB ID: 7M97), except for minor deviations of the side chains for Ile355 and an additional alternative conformation for Cys367 (**Figure** **1**A). The binding mode (BM) of the 4‐acyl pyrrole corresponds to that known for human BRDs (PDB ID: 7R5B).^[^ ^14^ ^]^ The aniline moiety in the binding pocket (BP) of *Pf*BDP1‐BRD adopts two 180° rotated conformations, but cannot displace the conserved water, as is the case for human BRDs^[^ ^14^ ^]^ (Figure S1A, Supporting Information). Initial models based on this structure and the affinity data suggested that small hydrophobic groups in ortho position should be beneficial. The resulting ligands (Table 1) confirmed this and showed that larger groups lead to weaker affinities. All molecules tested showed improved affinity over MPM3, but only methyl‐ and CF_3_‐groups (RMM2, 3.9 μM and RMM4, 2.9 μM, Table 1) led to better affinity than for the first hit (MPM6, 4.53 μM, Table 1). The co‐crystal structure of *Pf*BDP1‐BRD in complex with RMM2 (PDB ID: 9HHD, Figure 1B) in comparison with *Pf*BDP1‐BRD‐MPM2 reveals a similar binding mode for both inhibitors (Figure S1B, Supporting Information). Unlike MPM2, the phenyl group of RMM2 adopts only one conformation, with the methyl group forming hydrophobic interactions with the side chains of Ile355, the gatekeeper Val419, and the seven‐membered ring. The phenyl ring is also tilted about 20° and is placed closer to the outermost conserved water.

**Table 1:** ITC characterization of synthesized compounds with PfBDP1‐BRD.

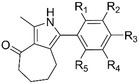						
Name	R_1_	R_5_	R_2_	R_4_	R_3_	*K*_D_ [μM]
MPM3	H	H	H	H	H	10.4
MPM6	Cl	H	H	NH_2_	H	4.53
MPM2	H	H	H	NH_2_	H	6.74
RMM1	Cl	H	H	H	H	5.81
RMM2	CH_3_	H	H	H	H	3.94
RMM4	CF_3_	H	H	H	H	2.94
RMM21	CH_3_	H	NHSO_2_CH_3_	H	H	2.76
RMM15	H	H	H	H	CONH_2_	4.49
RMM18	H	H	H	H	CN	4.54
RMM22	CH_3_	H	H	H	CN	2.34
RMM23	CH_3_	H	H	H	CONH_2_	1.24
RMM24	CH_3_	CH_3_	H	H	CN	1.22
RMM25	CH_3_	CH_3_	H	H	CONH_2_	0.896

In addition to the crystallization with *Pf*BDP1‐BRD, we successfully obtained co‐crystal structures with the more stable homolog *Pv*BDP1‐BRD, which we first achieved with RMM21 (PDB ID: 9HHB, Figure S1C, Supporting Information). The BP is indistinguishable from that of *Pf*BDP1‐BRD and, like *Pf*BDP1‐BRD‐MPM2, shows two conformations for Cys367 (Figure 1A). Apart from this, the BM of RMM4 is similar to *Pf*BDP1‐BRD‐RMM2, although the alignment of a fluorine of the CF_3_ group between the side chains of Ile355 and the gatekeeper Val419 shifts the phenyl ring even further toward the outermost conserved water (PDB ID: 9HHA, Figure S1D, Supporting Information).

Next, we investigated the influence of the *meta* position. Based on the affinity increase from MPM3 to MPM2, we assumed that hydrophilic groups improve affinity. Of the compounds tested (Table 1), the methyl variant (RMM6, 6.2 μM, Table 1) showed a moderate affinity increase compared to MPM2 (6.7 μM, Table 1), but only the sulfonamide‐substituted forms (RMM11, 4.7 μM and RMM13, 4.9 μM, Table 1) showed similar affinity as the first hit (MPM6, 4.53 μM, Table 1). Based on our models we assumed hydrogen bonds to the side chains of Gln365 and His354 for the sulfonamide variants. In the next step, we tried to enable hydrogen bonds via the para position, which should allow for similar hydrogen bonds, although unlike MPM2 not the whole phenyl ring would have to be rotated to align the functional group, and this should lead to better affinity. All hydrogen bond donors, as well as the cyanide group, resulted in better affinity than MPM2 and MPM3. This can be demonstrated particularly by the example of the differently methylated *p*‐amides, in which the affinity collapses for the dimethylation, as hydrogen bonds can no longer be formed. For instance, both a single methyl group at the para position and a dimethylated *para*‐amide result in a significant loss in affinity. (Table S2, Supporting Information). With the amide (RMM15, 4.49 μM, Table 1) and the cyanide (RMM18, 4.54 μM, Table 1) variants, we identified two molecules with slightly better affinity than the best *meta*‐variant.

Next, we attempted to combine some of the best ortho modifications with some of the best meta or para substituents (Table 1). Since our models suggested that meta and para substituents form similar hydrogen bonds, we refrained from testing these combinations. Of the combinations tested, three molecules showed improvement over their predecessors. The complex structure of *Pv*BDP1‐BRD‐RMM21 (PDB ID: 9HHB, Figure S1C, Supporting Information) largely resembles both the BP and the BM of *Pf*BDP1‐BRD‐RMM2 (Figure 1B), whereas the phenyl ring is oriented such that the meta substituent points in a single direction, unlike in *Pf*BDP1‐BRD‐MPM2, where it alternates between two orientations. In addition to the interactions described for *Pf*BDP1‐BRD‐RMM2, the structure of RMM21 confirmed the formation of hydrogen bonds to His354 or Gln365 with its sulfonamide, which is present in two conformations. The co‐crystal structures of RMM23 with *Pf*BDP1‐BRD (PDB ID: 9HH7, Figure S1E, Supporting Information) and *Pv*BDP1‐BRD (PDB ID: 9HH8, Figure 1C) are largely identical, although the *Pv*BDP1‐BRD structure is much better defined and resolved, with the only difference being the visibility of two conformations of GLN351. BP and BM are very similar to *Pf*BDP1‐BRD‐RMM2, with most of the methyl group orientated the same way but also with a much smaller proportion rotated by 180°. The amide does not appear to interact directly with the BP in the crystal, but this does not have to be the case in solution. Notably, His354 forms a hydrogen bond to a symmetry equivalent and may be no longer available. In the next step, we tested the possibility of introducing a second *o*‐methyl group. Based on the two conformations of RMM23 in the *Pf*BDP1‐BRD co‐crystal structure, this should be possible for RMM23 and RMM22, but not for RMM21 (compared to RMM20). The resulting ligands, RMM24 and RMM25 (Table 1), both show an improvement in affinity based on deteriorating enthalpy (ΔH), with simultaneously stronger improvement in entropy (ΔS) (Figure ITC, Supporting Information). This indicates that no new preferential interactions are formed, but the rotation to align the methyl group, that is necessary for RMM23, is not required. This is further supported by the structure of *Pv*BDP1‐BRD‐RMM25 (PDB ID: 9HGF, Figure 1D), which shows an unchanged BM and BP compared to *Pv*BDP1‐BRD‐RMM23 (Figure 1C).

The activity of the compounds RMM23 and RMM25 was tested against in vitro blood stage *P. falciparum* cultures of the wild type strains 3D7 and NF54, as well as against the multidrug resistant K1 strain (chloroquine, pyrimethamine, and sulfadoxine resistant). The BRDi compounds had modest activity against all three strains (**Table** **2**). RMM25 showed the lowest half maximal effective concentration (EC50) against the K1 strain, whereas RMM23 was most active against NF54 (Table 2).

**Table 2:** Dose response of *P. falciparum* isolates to BD inhibitors.

Compound	3D7	NF54	K1
	EC50 [μM]	EC50 [μM]	EC50 [μM]
RMM23	18.33 (±2.93)	13.96 (±3.25)	20.9 (±15.37)
RMM25	19.03	12.25	7.35 (±6.51)

Data presented as mean and SD (standard deviation) from 1 to 3 biological replicates.

Treatment of synchronous ring‐stage 3D7 and NF54 parasite cultures with RMM23 resulted in significantly reduced growth after 48 h of treatment, indicating that the compound blocked parasite development within the same IDC when treatment was initiated (Figure S2A,B, Supporting Information). Morphologically, RMM23 treated parasites developed normally into trophozoites by 24 h, but then failed to undergo DNA replication and schizogony. Conversely, treatment commenced in the trophozoite stage resulted in normal schizogony, but a block in egress and reinvasion within 24 h, causing a greatly diminished number of ring‐stage infected erythrocytes relative to the DMSO control (Figure S1C,D, Supporting Information). These results are consistent with those obtained by conditional KD of *Pf*BDP1^[^ ^12^ ^]^ and demonstrate that RMM23 can block parasite replication rapidly within the same IDC.

**Figure 2:**
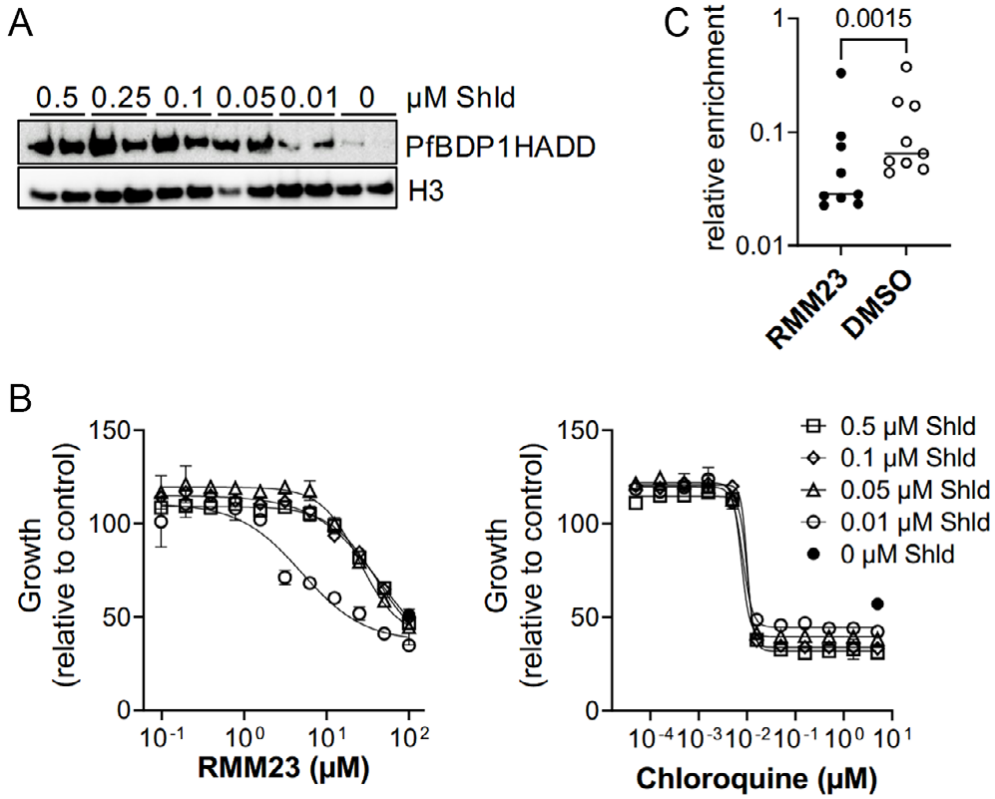
Target validation of RMM23. A) Western Blot analysis of 3D7::*Pf*BDP1HADD parasites cultivated in the presence of Shield1 (Shld) in concentrations ranging from 0 to 500 nM over 72 h in duplicates. *Pf*BDP1HADD was detected by anti‐HA antibodies. Histone 3 (H3) was probed as a loading control. B) Dose–response assay of 3D7::*Pf*BDP1HADD parasites to RMM23 (left) and chloroquine (right). Susceptibility to RMM23 increases under conditions of partial *Pf*BDP1 KD with 10 nM Shield1. Growth was determined relative to DMSO controls for each condition after 72 h of culture. *N* = 4 replicates. C) ChIPqPCR assay of *Pf*BDP1 target regions (*N* = 9) in 3D7::*Pf*BDP1HA parasites cultivated for 3 h in the presence of 50 μM RMM23 or DMSO as a control. Binding of *Pf*BDP1HA to target loci was calculated as enrichment relative to histone H3 ChIP performed in parallel. Shown are individual values and median of one representative experiment with technical duplicates. Paired t‐test, *p* = 0.0015.

To validate whether the compounds indeed target the BRD of *Pf*BDP1, we utilized a conditional KD system to modulate the expression levels of *Pf*BDP1 in situ.^[^ ^15^ ^]^ In the 3D7::*Pf*BDP1HADD line, *Pf*BDP1 is fused at its C‐terminus to a ligand‐regulatable FKBP destabilization domain (DD) that targets the fusion protein for proteasomal degradation but can be stabilized by the Shield1 ligand, and 3xHA epitope tags for detection ^11^. Titration of Shield1 from 500 to 10 nM resulted in diminished *Pf*BDP1 levels that still supported normal parasite growth (**Figure** **2**A). The conditional KD parasites (10 nM Shld1) exhibited increased sensitivity against RMM23, which was reflected by a leftward shift in the dose–response curve compared to standard conditions (500 nM Shld1) (Figure 2B). In contrast, the EC50 of chloroquine was unchanged. This observation is consistent with RMM23 acting directly on *Pf*BDP1. In contrast, the parasites only showed moderately increased sensitivity to RMM25 in this assay (Figure S3, Supporting Information), indicating that RMM25 may preferentially target the BRD of another *Pf*BDP.

*Pf*BDP1 is recruited to promoters of invasion‐related genes by binding with its BRD to acetylated histones.^[^ ^12^ ^]^ To examine whether RMM23 inhibits chromatin binding of *Pf*BDP1‐BRD, we performed ChIP of *Pf*BDP1HA in schizont stage parasites cultivated for 3 h with 50 μM RMM23 or DMSO control. Analysis of several known *Pf*BDP1 target loci by qPCR revealed significantly diminished enrichment of *Pf*BDP1 in the presence of RMM23 (Figure 2C), further supporting *Pf*BDP1‐BRD as the main target of RMM23 in *P. falciparum* parasites.

## 3 Conclusion

In summary, we developed inhibitors targeting *Pf*BDP1, an essential bromodomain protein in *P. falciparum*, and solved the first crystal structures of *Pf*BDP1‐BRD and *Pv*BDP1‐BRD with these inhibitors. Using in vitro assays combined with conditional KD of *Pf*BDP1, we identified RMM23 as a *Pf*BDP1‐specific inhibitor that demonstrated effective binding and inhibition of parasite growth. These findings highlight the potential of therapeutically targeting BRD proteins in malaria parasites and suggest RMM23 as a promising candidate for further optimization toward clinical development.

## Conflict of Interest

The authors declare no conflict of interest.

## Author Contributions

**Marius Amann**: conceptualization (equal); data curation (lead); formal analysis (equal); investigation (lead); methodology (lead); validation (lead); visualization (lead); writing—original draft (lead); writing—review & editing (equal). **Robin Warstat**: conceptualization (equal); formal analysis (equal); investigation (lead); methodology (lead); writing—review & editing (equal). **Kay Kristin Rechten**: data curation (equal); investigation (lead); visualization (supporting); writing—review & editing (supporting). **Philip Theuer**: investigation (supporting); methodology (supporting); writing—review & editing (supporting). **Magdalena Schustereder**: methodology (supporting); writing—review & editing (supporting). **Sophie Clavey**: investigation (supporting); methodology (supporting); writing—review & editing (supporting). **Bernhard Breit**: resources (equal); supervision (equal); writing—review & editing (supporting). **Oliver Einsle**: resources (equal); supervision (equal); writing—review & editing (supporting). **Martin Hügle**: conceptualization (equal); data curation (supporting); formal analysis (equal); investigation (supporting); methodology (equal); validation (equal); writing—original draft (lead); writing—review & editing (equal). **Michaela Petter**: conceptualization (equal); investigation (supporting); methodology (supporting); supervision (equal); validation (supporting); writing—original draft (equal); writing—review & editing (equal). **Stefan Günther**: conceptualization (equal); project administration (lead); supervision (equal); writing—original draft (supporting); writing—review & editing (equal). **Marius Amann** and **Robin Warstat** contributed equally to this work.

## Supporting information

Supplementary Material

## Data Availability

The data that support the findings of this study are available in the supplementary material of this article.
